# Signaling through NOD-2 and TLR-4 Bolsters the T cell Priming Capability of Dendritic cells by Inducing Autophagy

**DOI:** 10.1038/srep19084

**Published:** 2016-01-12

**Authors:** Nargis Khan, Aurobind Vidyarthi, Susanta Pahari, Shikha Negi, Mohammad Aqdas, Sajid Nadeem, Tapan Agnihotri, Javed N. Agrewala

**Affiliations:** 1 CSIR-Institute of Microbial Technology, Chandigarh-160036, India

## Abstract

T cells play a cardinal role in mediating protection against intracellular pathogens like *Mycobacterium tuberculosis (Mtb)*. It is important to understand the factors that govern the T cell response; thereby can modulate its activity. Dendritic cells (DCs) are the major player in initiation and augmentation of T cell response. Targeting DCs to induce their optimum maturation and activation can lead to a better T cell response. Interestingly, we observed that combinatorial signaling of DCs through NOD-2 and TLR-4 fortified better yield of IL-12p40/70, IL-6 and IFN-γ and upregulated the expression of CD40, CD80 and CD86 costimulatory molecules. Further, we noticed improved phagocytic capabilities of DCs. Furthermore, NOD-2 and TLR-4 induced autophagy in DCs, which enhanced the activation of T cells. This study signifies that NOD-2 and TLR-4 exhibit synergism in invigorating the activity of DCs. Consequently, this strategy may have significant immunotherapeutic potential in bolstering the function of DCs and thus improving the immunity against pathogens.

Host defense against *Mycobacterium tuberculosis (Mtb)* requires establishment of Th1 and Th17 immunity to ultimately eliminate this pathogen^1^. Initiation of T cells response requires three signals i) TCR-MHC-peptide complex; ii) costimulatory molecules; iii) proinflammatory cytokines^2^. Dendritic cells (DCs) are most potent antigen presenting cells (APCs) that deliver all the 3 signals and determine the fate (activation/anergy) of naive T cells^3^. However, it is important to mention that only mature DCs efficiently drive the activation and clonal expansion of T cells; consequently endures the immunity^4^. In essence, maturation and activation of DCs is a fundamental step for effector T cell response.

Toll-like receptors (TLRs) play an essential role in DCs maturation and activation^5^,^6^. Although receptors, such as TLR-2, TLR-4 and TLR-9 have been implicated in inducing the innate response against *Mtb* but their role seems to be limited^7^. Other innate receptors *viz* nucleotide binding oligomerization domain (NLRs), C-type lectin receptors (CLRs), etc., may contribute in recognition of pathogens like *Mtb* and mounting adaptive immune response against them. Mice deficient for NOD-2 showed impaired cytokine production by macrophages and DCs after *Mtb* infection^8^. Further, NOD-2 receptor has also been shown to amplify the TLRs signal. NOD-2 acts in synergy with TLRs and augments the release of proinflammatory cytokines by DCs^9^. In addition, it augments the bactericidal activity of DCs. This indicates that synergistic signaling through TLRs and NOD-2 may contribute in promoting adaptive immunity.

Autophagy plays a vital role in *Mtb* protection^10^,^11^. It targets the antigen to lysosomes for degradation and delivers anti-microbial peptides to *Mtb* harboring compartments. Simultaneously, it prevents the excessive inflammatory reaction in the host^11^. Further, autophagy enhances the antigen presenting ability of DCs to T cells^12^,^13^. Taking into consideration these facts, we were curious to monitor whether NOD-2 and TLR-4 signaling acts in concert to improve the functionality of DCs. Further, whether these DCs acquire greater ability to activate T cells and mechanism involved in this phenomenon. Interestingly, we observed that NOD-2 and TLR-4 triggering augments level of autophagy in DCs, which in turn amplify the T cell response.

## Results

### N_2_T_4_ stimulation of DCs enhances cytokine releases

**Figure 1:**
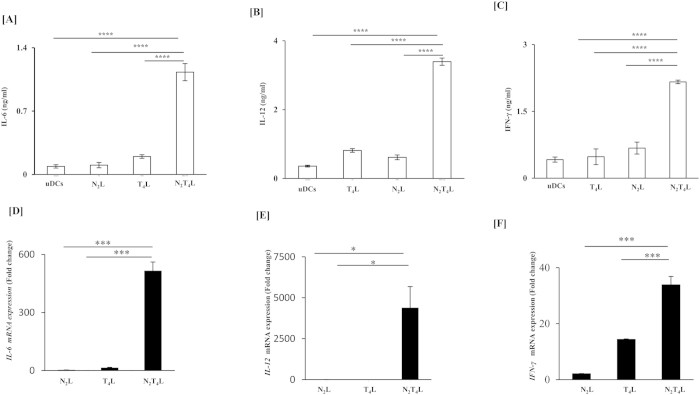
Cumulative signaling through N_2_T_4_ induces and enhances the release of IL-6, IL-12p40/70 and IFN-γ. DCs were stimulated through N_2_T_4._ The controls were elicited *via* T_4_ or N_2_ for 24 h. Later, culture SNs were assessed for release of (**A**) IL-6; (**B**) IL-12p40/70; (**C**) IFN-γ cytokines by ELISA. The ‘x axis’ signifies concentration of TLR-4L (5 ng/ml) and NOD-2L (10 μg/ml). Graphs depict mRNA expression of (**D**) IL-6; (**E**) IL-12p40; (**F**) IFN-γ relative to untreated controls by RT-qPCR. Data shown as mean ± SD are representative of two independent experiments. *p < 0.05, ***p < 0.001, ****p < 0.0001.

Initiation of immune response is critically dependent on the activation of DCs. This process starts with the release of cytokines. We observed that DCs triggered through TLR-4 showed dose dependent increase in the release of IL-6 (Fig. S1). However, NOD-2 triggering showed remarkably lesser production of IL-6, compared to TLR-4. Interestingly, combinatorial signaling through TLR-4 and NOD-2 (N_2_T_4_) exhibited synergistic impact and significantly enhanced the yield of IL-6 (p < 0.0001), IL-12p40/70 (p < 0.0001) and IFN-γ (p < 0.0001) compared to controls (N_2_L or T_4_L) (Fig. 1A–C). This observation related to IL-6, IL-12 and IFN-γ was further substantiated at mRNA level by RT-qPCR (Fig. 1D–F). Noteworthy, optimum release of IL-6 was observed at 10 μg/ml of N_2_L and 5 ng/ml concentration of T_4_L (Fig. S1A, B). Therefore, these doses were selected for all the experiments. To rule out the possibility of any contaminating cells in the results, DCs isolated by MACS showed 95% purity. These DCs were triggered through N_2_T_4_ and release of IL-12p40/70 was estimated in the culture SNs (Fig. S2A–D). We observed the similar pattern in the production of IL-12p40/70, as was noticed with cultured DCs (Fig. S2D, Fig. 1B).

### Signaling delivered through N_2_T_4_ induces maturation and activation of DCs

**Figure 2:**
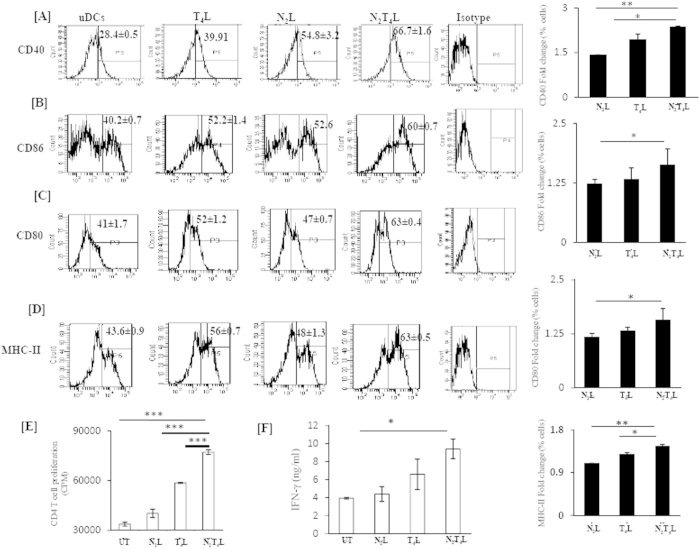
N_2_T_4_ triggers the maturation of DCs. The DCs were stimulated through N_2_T_4_ and controls *via* N_2_ or T_4_ for 24 h. Later, cells were stained for (**A**) CD40; (**B**) CD86; (**C**) CD80; (**D**) MHCII. Number in the inset of flowcytometry histograms (left panel) indicates the percent positive population and bar graph (right panel) depicts the percent population. Fold change was calculated with respect to untreated control. N_2_T_4_ stimulated DCs were γ-irradiated and co-cultured with purified CD4 T cells in 96 well plate coated with anti-CD3 Ab for 48 h. (**E**) Proliferation of CD4 T cells was assessed by thymidine incorporation; (**F**) IFN-γ was quantified in the culture SNs by ELISA. Data shown as mean ± SD are representative of two independent experiments. *p < 0.05, **p < 0.01, ***p < 0.001.

Maturation of DCs involves upregulation of expression of MHC-II and costimulatory molecules^14^. Intriguingly, we noted that signaling delivered through N_2_T_4_ augmented the expression of CD40 (p < 0.01), CD86 (p < 0.05), CD80 (p < 0.05) and MHC-II (p < 0.01), when compared to untreated DCs (uDCs) or treated with N_2_L or T_4_L (Fig. 2A–D). Similar results were noted with MACS purified DCs (Fig. S2E, F).

Next, we studied the potential of N_2_T_4_ triggered DCs to activate T cells. N_2_T_4_ activated DCs were co-cultured with anti-CD3 stimulated CD4 T cells. Interestingly, we observed that N_2_T_4_ stimulated DCs induced significant (p < 0.001) increase in the proliferation of T cells (Fig. 2E). Further, it was noticed that T cells cocultured with activated DCs showed better production of IFN-γ (p < 0.05) than control cells (Fig. 2F). These data suggest that combinatorial signaling delivered through N_2_T_4_ showed synergism in activating T cells.

### N_2_T_4_ signaling substantially improved the phagocytosis competence of DCs

**Figure 3:**
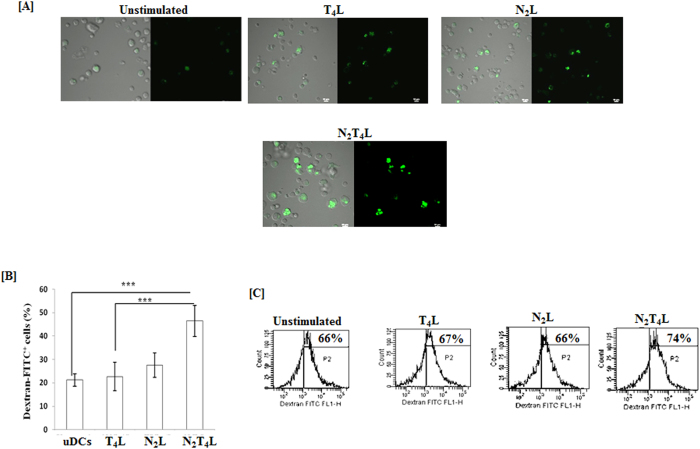
DCs stimulated through N_2_T_4_ efficiently phagocytosed the antigen. N_2_T_4_ activated DCs were incubated with dextran-FITC for 2 h. Dextran uptake by cells was assessed through (**A, B**) confocal microscopy (60X) and bar graph depicts the number of dextran-FITC^+^ cells, selected from 5-6 different fields; (**C**) flowcytometry Number in the inset of flowcytometry histograms indicates percentage of dextran-FITC^+^ cells. Data shown as mean ± SD are representative of two independent experiments. ***p < 0.001.

DCs is reasonably critical in capturing antigens. We observed noteworthy increase in the antigen uptake by N_2_T_4_ triggered DCs than controls. This was evidenced by higher antigen uptake by confocal microscopy (Fig. 3A, B). Further, these results were corroborated by flowcytometry data by significant (p < 0.0001) increase in the dextran-FITC uptake (Fig. 3C). These experiments suggest that N_2_T_4_ stimulation of DCs exhibited remarkable synergism between both the molecules in bolstering the antigen uptake by DCs.

### N_2_T_4_ stimulation induces autophagy in DCs

**Figure 4:**
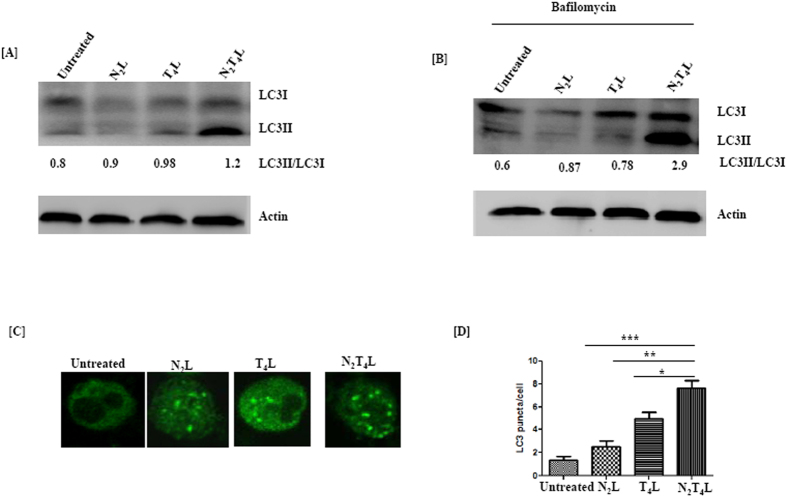
Signaling through N_2_T_4_ induces autophagy in DCs. DCs were stimulated through N_2_T_4_. Later, induction of autophagy was measured by conversion of LC3I to LC3II by Western blotting in the (**A**) absence; (**B**) presence of bafilomycin. (**C**) Puncta formation using immuno-fluorescent technique has been shown and (**D**) results (mean ± SD) are expressed as bar diagram. Puncta was enumerated taking into consideration 5-6 different fields, with 3-4 cells per field. Data shown are representative of two independent experiments.

Autophagy mediates the intracellular killing of bacteria by targeting antigen to lysosomal degradation pathway^15^. Further, it also contributes in the presentation of antigens *via* MHC class I and II pathways. We observed that N_2_T_4_ stimulation of DCs showed the increment in the conversion of LC3I to LC3II, which are markers for autophagy (Fig. 4A). Further, N_2_T_4_L stimulation showed higher accumulation of LC3II in the presence of bafilomycin, which blocked the fusion of autophagosome with lysosome and thereby preventing the degradation of LC3II (Fig. 4B). Furthermore, we corroborated these results by demonstrating puncta formation through immunofluorescence staining (Fig. 4C,D).

### N_2_T_4_ activated DCs acquired enhanced capability to prime naive T cell

**Figure 5:**
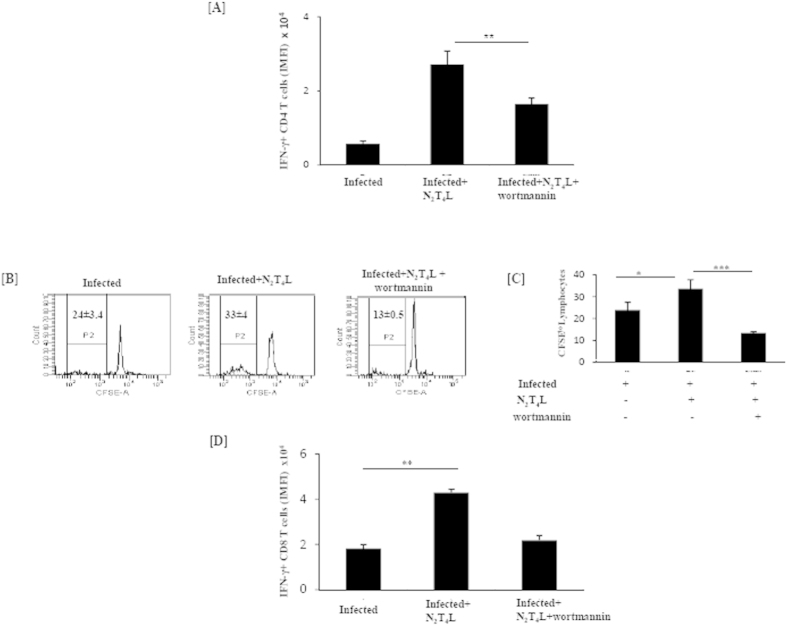
N_2_T_4_ stimulation induces autophagy in DCs that bolsters the T cells response. DCs pre-treated with wortmannin were infected with *Mtb* for 4 h. Later, cells were extensively washed and stimulated through N_2_T_4_ for 24 h. These DCs were adoptively transferred into mice. After 5d, lymphocytes were isolated from draining LNs and *in vitro* cultured for 48 h with PPD. (**A,B**) Intracellular expression of IFN-γ was detected in CD4 T cells and CD8 T cells by flowcytometry and data expressed as IMFI in the form of bar digram. IMFI is calculated by multiplying the frequency (% positive) of cells expressing a particular cytokine with the mean fluorescence intensity (MFI) of that population. (**C,D**) lymphocytes proliferation was determined by CFSE-dye dilution assay. Number in insets indicates CFSE^lo^ lymphocytes. Decrease in the (**A,B**) IMFI of IFN-γ^+^ cells and (**C,D**) proliferation by wortmannin indicates that the phenomenon is autophagy dependent. Data shown as mean ± SD are representative of two independent experiments. *p < 0.05, **p < 0.01, ***p < 0.001.

To confirm the potency of N_2_T_4_ activated DCs to prime naive T cells, *Mtb* infected DCs triggered *via* N_2_T_4_ were adoptively transferred into mice. After 5d, it was ascertained that CD4 T cells or CD8 T cells primed by N_2_T_4_ activated DCs, efficiently triggered the IFN-γ (p < 0.001) release, as compared to infected or untreated DCs after *in vitro* stimulation with PPD (Fig. 5A,B, Fig. S3A,C). The lymphocytes isolated from the mice that were adoptively transferred with infected DCs were cultured *in vitro* with PPD.

Earlier, we have demonstrated that N_2_T_4_ activated DCs showed augmented autophagy. Autophagy is a bactericidal phenomenon but simultaneously it is known to enhance T cell response^15^,^16^. Therefore, we were curious to study whether N_2_T_4_ induced autophagy in DCs exhibits any effect on priming of naive T cells. To confirm this, DCs were incubated with wortmannin to block autophagy, prior to *Mtb* infection followed by stimulation through N_2_T_4_. We observed that such DCs showed significant (p < 0.001) decline in the secretion of IFN-γ by CD4 T and CD8 T cells (Fig. 5A,B, Fig. S3A–D). We also used another autophagy inhibitor 3MA, to further validate our results (Fig. S3E,F). It is important to mention that the effect of wortmannin used as an autophagy inhibitor showed no effect on the viability of DCs or in the level of costimulatory molecules CD40 and CD86 expressed on DCs (Fig. S4A–C). Further, we observed significant (p < 0.001) decrease in the proliferation of lymphocytes on *in vitro* challenge with PPD (Fig. 5C,D). The antigen specificity was proved by *in vitro* stimulation of lymphocytes isolated from the mice adoptively transferred with *Mtb* infected DCs treated with N_2_T_4_L with OVA, a non *Mtb* antigen. Overall the results demonstrate that N_2_T_4_ induced autophagy enhances the DCs capacity to activate T cells.

## Discussion

Dendritic cells are the major player in the generation of effective T cell responses^17^. Importantly, DCs efficacy depends on many variables, especially maturation status and efficient antigen presentation to naive T cells. However, signaling of DCs through surface receptors in particular, improve their potency in enhancing adaptive immunity^18^,^19^. Ligation of CD40 on DCs triggers the production of IL-12 and augments their T cell stimulatory capacity^20^. Further, it has been documented that signaling *via* TLR-4 amplifies the outcome of CD40 response. Cumulative signaling of CD40 and TLR-4 enhances the production of IL-12 by DCs and improve their anti-tumor efficacy^21^. These DCs stimulated T cells against tumor-associated antigens. It suggests that crosstalk between NOD-2 and TLR-4 stimuli may lead to a better performance of DCs; as has been noticed in the case of better release of cytokines, upregulation of costimulatory molecules and phagocytic activity. However, the role of such DCs in the activation of T cells has not yet been studied. Therefore, we thought that cumulative signaling through N_2_T_4_ may be imperative in bolstering DCs functions to improve T cells response. We selected N-glycolyl MDP as NOD-2 agonist; since it exhibits 10–100 fold more potent immunogenicity than the commonly studied N-acetylated MDP^22^. We used LPS as a source of TLR-4 ligand. Recently, Food and Drug Administration (FDA) has approved its use in future medicines, which has opened new avenues to harness its remedial potential^23^.

In the current study, delivering combinatorial signals through N_2_T_4_ to DCs led to the emergence of following interesting findings: i) enhanced activation and maturation of DCs; ii) augmented phagocytosis by DCs; iii) increased autophagy; iv) improved capability of DCs to prime naive T cells. Intriguingly, we observed that signaling through N_2_T_4_ induced robust release of IL-6, IL-12p40/70, and IFN-γ by DCs. Importantly, these cytokines play important role in the activation of not only naive T cells but also helps in stimulating other cells responsible for sustaining immunity, including DCs. Further, IL-12 promotes the differentiation of naive CD4 T cells to Th1 subtype. Th1 cells perform cardinal function to protect against intracellular pathogens like *Mtb*^24^. IFN-γ is well known cytokine for the induction of the expression of MHC-I and MHC-II molecules.

It is well established that the expression of costimulatory molecules is critical for the activation of T cells^18^,^19^,^25^,^26^. Interestingly, we also observed the upregulation of costimulatory molecules such as CD40, CD80 and CD86 on N_2_T_4_ activated DCs. Optimum expression of costimulatory molecules on DCs is exceedingly essential in deciding the activation or anergy of naive T cells^27^.

To prime T cells, the primary function of the immature DC is to capture antigen. Importantly, N_2_T_4_ triggered DCs displayed better phagocytic capacity. After antigen is captured, it is processed by exogenous and endogenous pathways^28^. In addition to classical pathways, autophagy has also been reported to enhance the antigen presentation by APCs to CD4 and CD8 T cells^29^,^30^. It is worth to mention here, that N_2_T_4_ stimulation of DCs augments autophagy. Autophagy plays a critical role in elimination of pathogens by targeting them to lysosomal degradation pathway^31^. Importantly, we observed that blocking of autophagy induced *via* N_2_T_4_ signaling in DCs suppresses IFN-γ release by T cells. Thus this study opens new avenue of exploring immunomodulators to invigorate the potency of DCs to prime naive T cells.

## Material and Methods

### Animals

C57BL/6 mice, 6–8 weeks were procured from the Institute of Microbial Technology (IMTECH), Chandigarh, India.

### Ethics statement

All experiments were approved by the Institutional Animal Ethics Committee of Institute of Microbial Technology and performed according to the National Regulatory Guideline issued by Committee for the Purpose of Supervision of Experiments on Animals (No. 55/1999/CPCSEA), Ministry of Environment and forest, Govt. of India.

### Antibodies and reagents

All standard chemicals and reagents used in the study were purchased from Sigma (St. Louis, MO) and Abs and recombinant cytokines from BD Biosciences (San Diego, CA), unless and otherwise mentioned. TLR-4 ligand (LPS) and NOD-2 ligand (N-glycolyl MDP) were procured from Invivogen (San Diego, CA). Anti-mouse LC-3 Ab was obtained from Sigma (St. Louis, MO).

### Mycobacterial strain and antigens

*Mtb* strains (H37Rv, H37Ra) were provided by Dr. VM Katoch, National JALMA Institute for Leprosy and Other Mycobacterial Diseases, Agra, India. *Mtb* was cultured in Middlebrook 7H9 broth containing glycerol (0.2%) and Tween-80 (0.05%), supplemented with albumin, dextrose and catalase. The viability of the bacteria by colony forming units (CFUs) was determined by plating on Middlebrook 7H11 medium supplemented with oleic acid, albumin, dextrose and catalase.

### Culture of bone marrow derived DCs and their stimulation through NOD-2 and TLR-4 (N_2_T_4_)

Bone marrow derived DCs were cultured according to Lutz *et al*.^32^. Briefly, bone marrow cells (BMCs) were flushed aseptically from femurs and tibia. For DC cultures, cells were grown in RPMI 1640 (Invitrogen, Life Technologies, Eugene, OR) containing FCS-10% (GIBCO, Grand Island, NY) supplemented with penicillin (100 U/ml), streptomycin (100 mg/ml), and L-glutamine (100 mM), and granulocyte-macrophage colony-stimulating factor (GMCSF) (2 ng/ml) and murine rIL-4 (4 ng/ml) for 6d. Cultures were maintained in a humidified atmosphere, CO_2_ (5%) at 37 °C. The medium was replenished on 3d. Later, DCs were harvested, washed and stimulated for 24 h with N-glycolyl MDP (10 μg/ml) and LPS (5 ng/ml) as ligands of NOD-2 (N_2_L) and TLR-4 (T_4_L), respectively. These doses were selected on the basis of optimum secretion of IL-6 observed during checker board titration of the doses of NOD-2 and TLR-4 (Fig. S1). Bone marrow derived DCs were purified through magnetic associated cell sorting (MACS) as per the manufacturer’s instructions (BD Biosciences, San Diego, CA). Later, purified DCs (95%) were stimulated through N_2_T_4_, as described above.

### Cytokines estimation by ELISA

Cytokines IL-6, IL-12p40/70 and IFN-γ were detected in culture SNs at indicated time point by standard ELISA, according to manufacturer’s instructions (BD Biosciences, San Diego, CA).

### Flowcytometric analysis for the expression of activation markers

DCs stimulated with N_2_T_4_ and controls with N_2_ and T_4_ for 24h were harvested and resuspended in staining buffer (2% FCS, 2 mM NaN_3_ in PBS). To block non-specificity, cells were first incubated with Fc block (anti-CD16/32 Ab) for 25 min/4 °C. The cells were washed and then stained with fluorochrome conjugated Abs specific for CD80, CD86, CD40 and MHC-II and the control cells with isotype-matched Abs for 30 min/4^o^C. Cells were washed and fixed with paraformaldehyde (1X). Data were collected using FACS ARIA II and analyzed with BD DIVA software.

### *In vitro* proliferation of T cells

DCs (C57BL/6) were stimulated through N_2_T_4_ for 24 h. Later, DCs were γ-irradiated and cocultured with MACS sorted CD4 T cells (C57BL/6) in 96w plate coated with anti-CD3 Ab (2 μg/ml) for 48 h. IFN-γ was detected in the culture SNs by ELISA. Proliferation was assessed by incorporating thymidine in the cultures for subsequent 16 h. The radioactivity incorporated was measured by β–scintillation counting.

### Antigen uptake

DCs were stimulated through N_2_T_4_ and controls with N_2_ and T_4_ for 24 h. Later, activated DCs were incubated with dextran-FITC (1 mg/ml) for 2 h. It was followed by extensive washing with PBS. The cultures were fixed with paraformaldehyde and confocal microscopy (NIKON A1) was performed.

### Western Blotting

DCs were stimulated either through N_2_T_4_ or controls *via* N_2_ or T_4_ in the presence or absence of bafilomycin (100 nM) or 2 h. Later, cells were harvested, washed, and lysed in lysis buffer (RIPA buffer, protease and phosphatase inhibitor cocktail). In SNs, proteins were estimated and equal concentration was subjected to SDS-PAGE. After transfer to nitrocellulose membrane and subsequent blocking, the membranes were immunoblotted with Abs against LC3-I/ LC3II and actin as a loading control. Blots were developed using chemiluminescence kit (Amersham Pharmacia Biotech, Buckinghamshire, UK). Blots were scanned with the help of phosphoimager (Fujifilm, Tokyo, Japan) and image analysis was performed with MultiGuage software.

### Immunofluorescence staining

DCs were stimulated through N_2_T_4_ and controls *via* N_2_ or T_4_ for 4 h. Later, cells were harvested and fixed with 4X paraformaldehyde for 10–15 mins. It was followed by treatment with tween-20 (0.1%) for 15 sec. Cells were extensively washed with PBS. To block non-specific sites, DCs were incubated with BSA (5%) for 3 h, followed by rabbit anti-mouse LC3 Ab for 4 h. After 3X washing, cells were incubated with anti-rabbit FITC for 1 h. Cells were imaged under fluorescence microscopy.

### RT-qPCR for the quantification of IFN-γ, IL-6 and IL-12p40

Total RNA was isolated by trizol reagent from DCs stimulated through N_2_T_4_ or controls *via* N_2_ or T_4_ for 6 h, according to the manufacturer’s instructions (Invitrogen, Carisbad, CA). RNA was quantified with the help of NanoDrop spectrophotometer. A260/A280 ratio of all samples was in the range of 1.90 to 2.00. Intactness of RNA samples was determined with the help of formaldehyde denaturing agarose gel-electrophoresis. DNA contamination from RNA samples was removed by amplification grade DNase. Briefly, RNA samples (1 μg) were incubated with DNase (1U) for 15 min in the reaction buffer. After the incubation, DNase was terminated by stop solution. Further, the samples were heated to 70˚C/10 min to inactivate DNase activity. Results are represented in the form of re-expression (fold) relative to untreated controls. Analysis was done by comparative Ct method, whereas Ct values were normalized against house-keeping control actin. Using the comparative Ct method relative gene expression was calculated as 2^(-∆∆Ct)^, where ∆Ct = Ct (gene of interest)– Ct (normalizer = β-actin) and the ∆∆Ct = ∆Ct (sample)-∆Ct (calibrator). Calibrator was total RNA from lungs of placebo. RT-qPCR and data analysis was done by Realplex Master cycler (Eppendorf, Hamburg, Germany).

IFN-γ: Fwd 5^1^-CTAAGCAAGGACGGCGAAT-3^1^

Rev 5^1^-TTCCACACTGCACCCACTT-3^1^

β-actin: Fwd 5^1^-AGAGGGAAATCGTGCGTGAC-3^1^

Rev 5^1^-CAATAGTGATGACCTGGCCGT-3^1^

IL-6 Fwd 5^1^-GAGGATACCACTCCCAACAGACC-3^1^

Rev 5^1^-AAGTGCATCATCATCGTTGTTCATACA-3^1^

IL-12p40 Fwd 5^1^-GGAAGCACGGCAGCAGCAGAATA-3^1^

Rev 5^1^-AACTTGAGGGAGAAGTAGGAATGG-3^1^

### *In vivo* T cell response

DCs (3 × 10^6^ cells) infected with *Mtb* followed by stimulation through N_2_T_4_ were adoptively transferred (s.c) in mice. After 5d, mice were sacrificed and draining lymph nodes were isolated and single cell suspension was prepared^33^. Lymphocytes were cocultured with PPD (10 μg/ml) or OVA (10 μg/ml) for 48 h. Later, cells were treated with PMA (20 μg/ml) and ionomycin (1 μM) for 2 h followed by brefeldin for 3 h to detect IFN-γ expression through intracellular staining flowcytometer. Proliferation of lymphocytes in response to *in vitro* stimulation with PPD was assessed by carboxyfluoresceinsuccinimidyl ester (CFSE)-dye dilution by flowcytometry. To block autophagy, prior to *Mtb* infection DCs were treated with wortmannin (200 nM) and 3-Methyl adenine (10 mM) for 4 h^33^.

### Statistical analysis

Data were examined by one way analysis of variance (ANOVA) with post Tukey-Kramer multiple comparisons test by using Graph Pad Prism software.

## Additional Information

**How to cite this article**: Khan, N. *et al*. Signaling through NOD-2 and TLR-4 Bolsters the T cell Priming Capability of Dendritic cells by Inducing Autophagy. *Sci. Rep*. **6**, 19084; doi: 10.1038/srep19084 (2016).

## Supplementary Material

Supplementary Information
