# Supplementary material for: Signaling through NOD-2 and TLR-4 Bolsters the T cell Priming Capability of Dendritic cells by Inducing Autophagy
Source: Sci Rep. 2016 Jan 12;6:19084. doi: 10.1038/srep19084 (PMC4709561; doi:10.1038/srep19084)

**Signaling through NOD-2 and TLR-4 Bolsters the T cell Priming Capability of Dendritic cells by Inducing Autophagy**

Nargis Khan, Aurobind Vidyarthi, Susanta Pahari, Shikha Negi, Mohammad Aqdas, Sajid Nadeem, Tapan Agnihotri and Javed N Agrewala*

CSIR-Institute of Microbial Technology, Chandigarh-160036, India

**Supplementary Figures**

Fig. S1. *N_2_T_4_ triggering of DCs augments the release of IL-6.* DCs were triggered through various combinations of doses of N_2_ and T_4_ for 24h. The controls were also included, where DCs were activated *via* N_2_ or T_4_. Later, release of IL-6 was detected in SNs. (B) Bar graph depicts the dose dependent release of IL-6 upon stimulation through N_2_ alone. The data represented as mean±SD are from 2 independent experiments.

Fig. S2. *CD11c enriched DCs showed the matured and activated phenotype upon stimulation through N_2_T_4_*. (A) The purity of bone marrow cells cultured in the presence of GM-CSF and IL-4 (BMDC) or (B) BMDCs further enriched by MACS was ascertained by the expression of CD11c and IA^b^ molecules by flowcytometry. (C) Bright field microscopy depicts the morphology of DCs at a 40X magnification. MACS sorted CD11c^+^ DCs were stimulated through N_2_T_4_ for 24h. (D) Release of IL-12 was estimated in the culture SNs through ELISA and bar graph represents the mean±SD from 2 experiments. (E, F) CD11c^+^ DCs were stained for CD86 and CD80 expression. The data shown in the flowcytometry dot plots or histograms are the percentage of the cells and bar diagram depicts the fold change of percent gated population with respect to untreated control. Data are represented as mean±SD from 2 experiments. *p<0.05, **p<0.01, ***p<0.001.

Fig. S3. *N_2_T_4_ stimulation of DCs induces autophagy that augments T cell response.* DCs pre-treated with wortmannin and 3MA were infected with *Mtb* for 4h. Later, DCs were extensively washed and stimulated through N_2_T_4_ for 24h. These DCs were adoptively transferred into mice. After 5d, lymphocytes were isolated from draining LNs from all the groups and *in vitro* cultured for 48h with PPD. The animals adoptively transferred with *Mtb* infected N_2_T_4_.DCs showed enhancement in the intracellular expression of IFN-γ in (A) CD4 T cells; (C) CD8 T cells. The specificity of IFN-γ induced autophagy was proved by its inhibition by (B, D) wortmannin and (E, F) 3MA in (B, E) CD4 T cells; (D, F) CD8 T cells by flowcytometry, respectively. (E, F) the control cells were cultured with a non-*Mtb* protein OVA to prove specificity that the upregulation in the expression of IFN-γ is in response to only *Mtb* antigens (PPD). Number in the inset of dot plots indicates the percentage of IFN-γ^+^ cells. Bar diagram depicts the IMFI for IFN-γ^+^ cells. IMFI is calculated by multiplying the relative frequency (% positive) of cells expressing a particular cytokine with the mean fluorescence intensity (MFI) of that population. Untreated samples indicate *Mtb* uninfected and N_2_T_4_ untreated DCs that were adoptively transferred into mice. The results shown as mean±SD are representative of 2 independent experiments. *p<0.05, **p<0.01, ***p<0.001.

Fig. S4. *Wortmannin showed no effect on the costimulatory molecules and viability of DCs.* DCs pretreated with wortmannin were infected with *Mtb* for 4h. After 24h of incubation, DCs were stained for the expression of (A) CD40; (B) CD86 and (C) viability was assessed by propidium iodide (PI) staining.


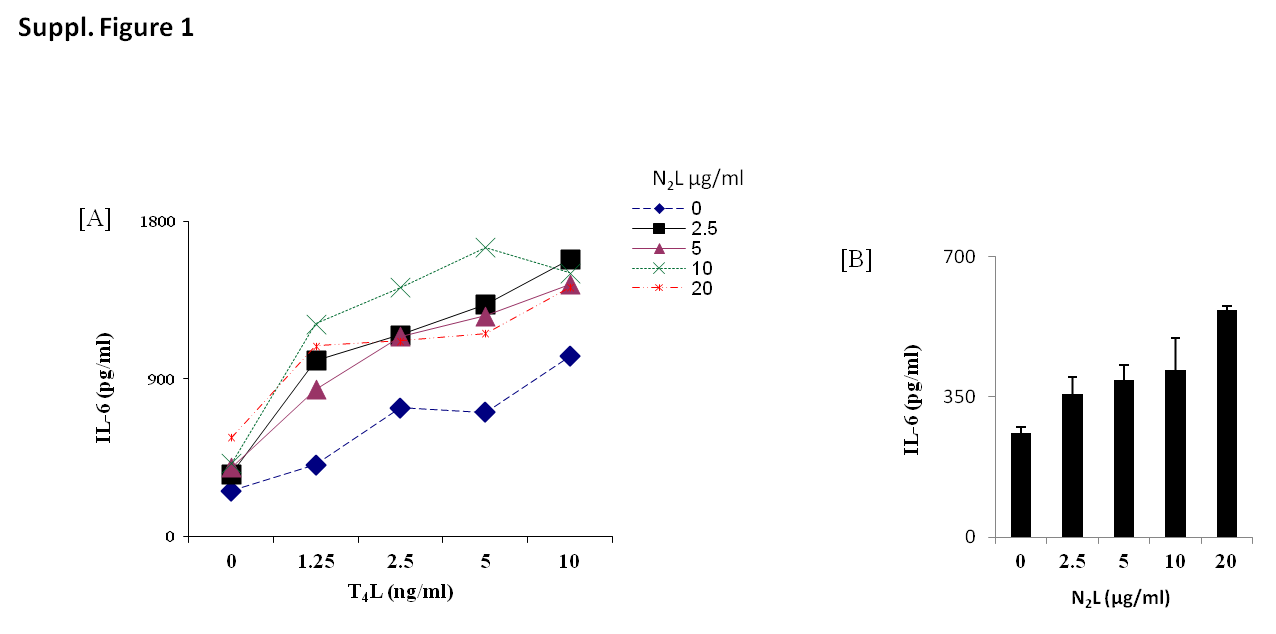


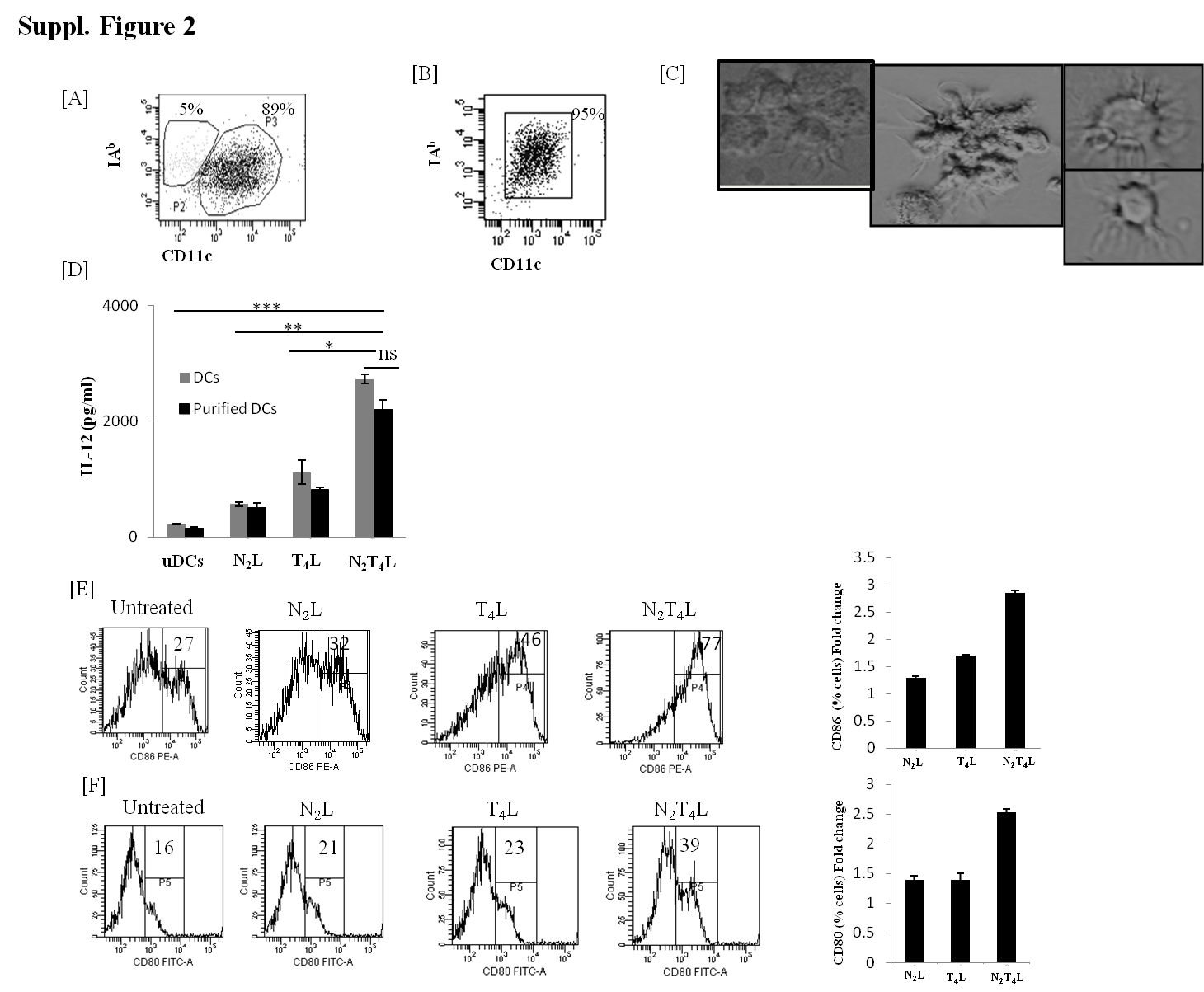


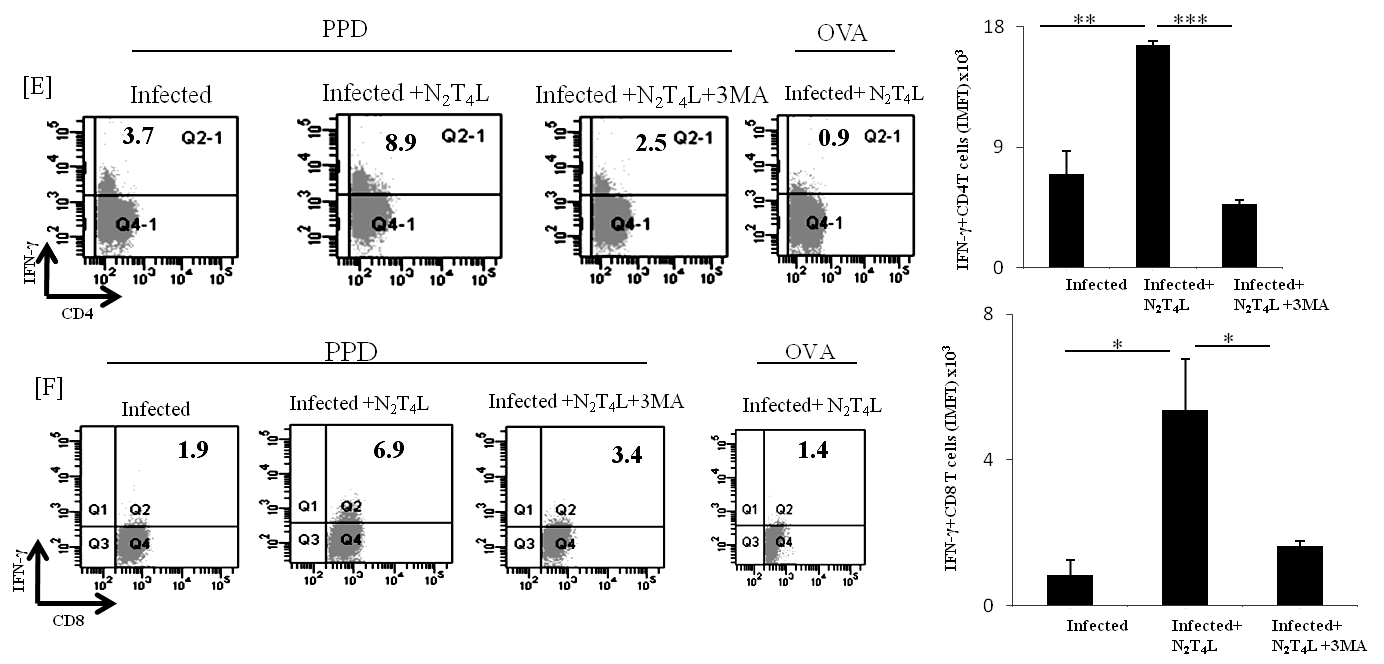


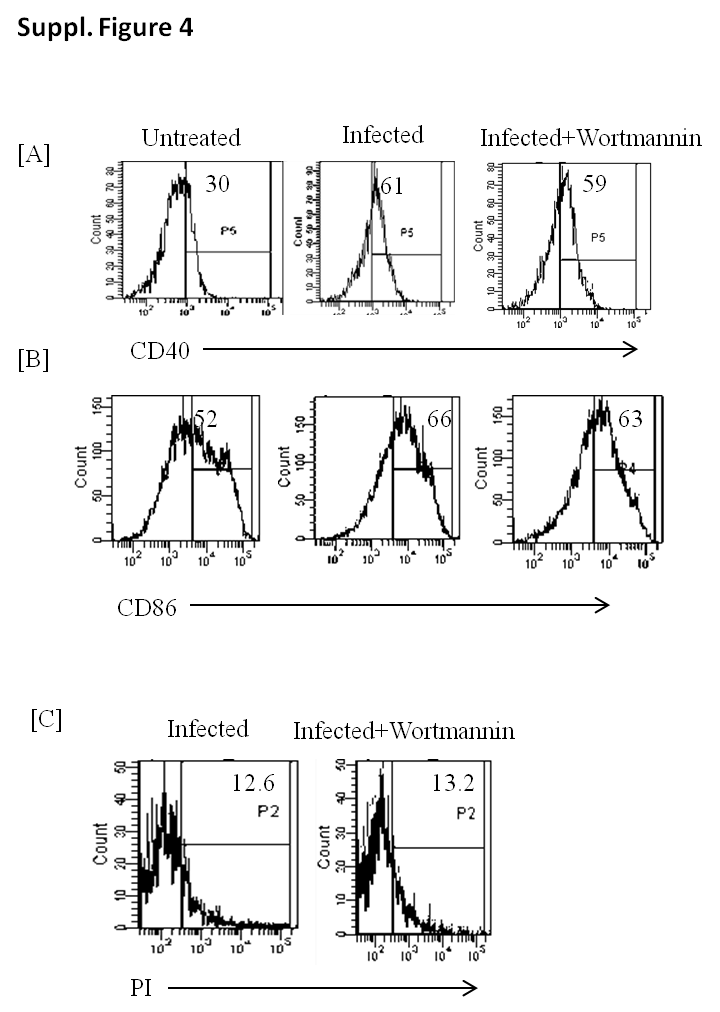

Supplement: Supplementary Information [file srep19084-s1.doc]
